# Physicochemical Properties of Tin and Neodymium Co-Doped Phosphate Glasses: Tuning the UV-Excited Nd^3+^ NIR Emission via Sn^2+^

**DOI:** 10.1021/acsorginorgau.5c00006

**Published:** 2025-05-02

**Authors:** José A. Jiménez, Dugan Hayes, Solaleh Farnia, Michael Vautier

**Affiliations:** † Center for Advanced Materials Science, Department of Biochemistry, Chemistry & Physics, 7604 Georgia Southern University, Statesboro, Georgia 30460, United States; ‡ Department of Chemistry, 4260 University of Rhode Island, Kingston, Rhode Island 02881, United States

**Keywords:** phosphate glasses, photoluminescence, physical properties, spectroscopy, structural properties, thermal properties

## Abstract

This work reports on various physicochemical properties and energy conversion processes in phosphate glasses containing Sn^2+^ and Nd^3+^ ions of interest for luminescence-based applications. The glasses were prepared by melting with 50P_2_O_5_-(49 – *x*)­BaO-1Nd_2_O_3_-*x*SnO (*x* = 0, 1.0, 3.0, 5.0, 7.0, and 9.0 mol %) nominal compositions and characterized by X-ray diffraction, ^119^Sn Mössbauer spectroscopy, density and related physical properties, Raman spectroscopy, differential scanning calorimetry, dilatometry, optical absorption, and photoluminescence (PL) spectroscopy. X-ray diffraction confirmed the noncrystalline nature of the glasses. The ^119^Sn Mössbauer evaluation allowed for estimating the relative amounts of Sn^2+^ and Sn^4+^ in the glasses, which showed that Sn^2+^ occurrence was favored. The densities showed variations without definite trends; additional physical parameters were then determined such as Sn^2+^-Nd^3+^ distances based on ^119^Sn Mössbauer results. The characterization by Raman spectroscopy showed no significant structural variation was induced as SnO replaced BaO. The thermal properties of the codoped glasses assessed were however found to be impacted mostly by Sn^2+^ at high nominal SnO contents. Absorption spectra supported consistent occurrence of Nd^3+^ ions among the codoped glasses. The PL evaluation showed that exciting Sn^2+^ centers in the UV (e.g., near 290 nm) results in near-infrared emission from Nd^3+^, which was maximized for SnO added at 5 mol %. The visible PL data were consistent with the presence of Sn^2+^ in the glasses and showed dips in the emission spectra, indicating the energy transfer to Nd^3+^ ions. The Nd^3+^ decay times were however similar among the different samples.

## Graphical abstract

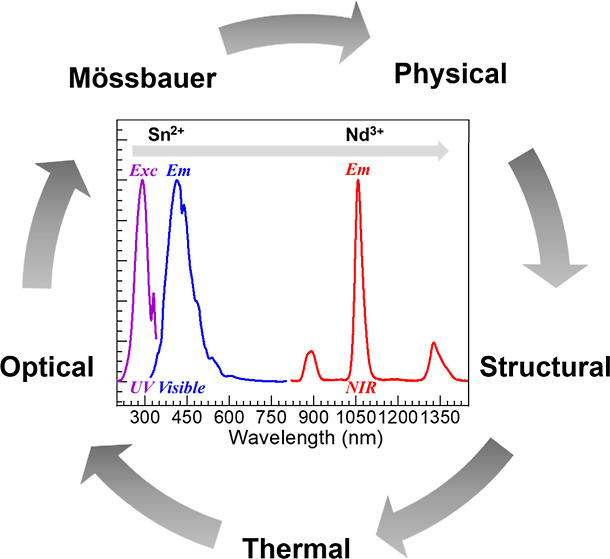

## 1 Introduction

Glasses doped with Nd^3+^ (f^3^) ions have been the subject of interest in optical technologies, with their near-infrared (NIR) emission being especially useful in high-power lasers.^1–11^ Hence, it has been a topic of inquiry to find ways to maximize the Nd^3+^ luminescence output and add versatility to the laser pumping scheme. One of the strategies commonly investigated to enhance Nd^3+^ NIR emission or provide alternate routes for nonresonant excitation is based on codoping with metal ions acting as sensitizers, including Cr^3+^,^12^ Mn^2+^,^13,14^ Sn^2+^,^15,16^ Cu^+^,^12,17^ and Ag^+^.^18,19^ Among these, the use of Sn^2+^ was first proposed as a sensitizer for Nd^3+^ ions in La-containing glass by Malashkevich et al.^15^ over three decades ago. Their pioneering work, however, focused exclusively on evaluating the optical properties of SnO-La_2_O_3_-Nd_2_O_3_ containing glasses.^15^ More recently, Bondzior and Lisiecki^16^ reported on the energy transfer processes involving Sn^2+^ and Nd^3+^ as well as Yb^3+^ ions in lanthanum borate glasses. The authors noticed that the Sn^2+^ → Nd^3+^ transfer efficiency was especially high, and further evaluations suggested that the interaction was of the dipole–dipole type.^16^ Nonetheless, although the work embarked on an extensive optical investigation that included temperature-dependent phenomena,^16^ questions remain with regards to the impact of tin on various physical, structural, and thermal properties of the glass. Hence, there is still room for holistically investigating the physicochemical properties of a variety of glass systems containing SnO and Nd_2_O_3_.

Divalent tin is also commonly utilized as a reductant to stabilize other redox active species in glasses.^17,18,20^ Apart from this use in formulations, its role as a Nd^3+^ NIR emission enhancer has been partially recognized in phosphate glasses co-doped with SnO/CuO^17,20^ and SnO/Ag_2_O.^18^ However, the sole effect of luminescent Sn^2+^ centers on Nd^3+^ ions in phosphate glasses remains largely unexplored. Phosphate-based glasses are specifically desirable for Nd^3+^-based lasing due to their favorable solubility, thermo-mechanical properties, and manufacturability.^1–5^ Divalent tin is also an interesting *n*s^2^-type center with blue-emitting character and has therefore been studied in phosphate glasses for light-emitting applications.^21–24^ Various studies have thus been devoted to assess exclusively the role of tin ions in enhancing the luminescent properties of phosphate glasses in the presence of various rare-earths such as Eu^3+^,^25,26^ Pr^3+^,^27,28^ Dy^3+^,^29^ Sm^3+^,^30^ Er^3+^,^31,32^ Gd^3+^,^33^ Yb^3+^,^16,34,35^ and Tb^3+^.^36,37^ Complementing this literature with new studies on SnO/Nd_2_O_3_ codoped phosphate glasses is then desired.

In this setting, we pursued a comprehensive experimental investigation of the effects of adding SnO at various concentrations on Nd^3+^-activated phosphate glasses of interest for optical applications. Considering the appeal of a simple fabrication method, material preparation was carried out by melting in ambient atmosphere, wherein the SnO concentration influences the resulting tin valences.^37^ The barium phosphate glass matrix used as host in prior studies^17,26,28–30^ was employed for SnO/Nd_2_O_3_ codoping, as it is considered adequate from fundamental and practical standpoints. The glasses were made with fixed Nd_2_O_3_ content at 1 mol %, which was found to be optimal in a recent investigation concerning 50P_2_O_5_-(50 – *x*)­BaO-*x*Nd_2_O_3_ (*x* = 0, 0.5, 1.0, 2.0, 3.0, 4.0 mol %) glasses.^11^ The amount of SnO was then varied while replacing BaO within the 50P_2_O_5_-(49 – *x*)­BaO-1Nd_2_O_3_-*x*SnO compositions with *x* = 0, 1.0, 3.0, 5.0, 7.0, and 9.0 mol % with the aim of identifying the amount of tin that enhances Nd^3+^ emission optimally. Comprehensive experimental evaluations were carried out using X-ray diffraction (XRD), ^119^Sn Mössbauer spectroscopy, densitometry, Raman spectroscopy, differential scanning calorimetry (DSC), dilatometry, UV–vis–NIR optical absorption, and photoluminescence (PL) spectroscopy. The results were then analyzed to link the different physicochemical parameters to the variation in absolute and relative Sn^2+^ and Sn^4+^ concentrations as assessed by ^119^Sn Mössbauer spectroscopy. The concentration of divalent tin for achieving the sensitized UV-excited NIR emission from Nd^3+^ ions attractive for optical devices is then finally endorsed.

## 2 Experimental Section

### 2.1 Glass Preparation

The glasses were prepared by melting under ambient atmosphere using as raw materials P_2_O_5_ (Thermo Scientific, 98%), BaCO_3_ (Thermo Scientific, 99.8%), Nd_2_O_3_ (Thermo Scientific, 99.99%) and SnO (Thermo Scientific, 99%). SnO was added at the expense of BaO targeting the 50P_2_O_5_-(49 – *x*)­BaO-1Nd_2_O_3_-*x*SnO (*x* = 0, 1.0, 3.0, 5.0, 7.0, and 9.0 mol %) nominal compositions. The different glasses prepared are summarized in Table 1 with the assigned glass codes. The amount of Nd_2_O_3_ being fixed at 1 mol % follows a previous work where this concentration was found to produce maximum PL in the 50P_2_O_5_-(50 – *x*)­BaO-*x*Nd_2_O_3_ (*x* = 0, 0.5, 1.0, 2.0, 3.0, 4.0 mol %) glass system.^11^ The raw materials were weighed in the appropriate quantities (making about 25 g batches), thoroughly mixed and melted in porcelain crucibles at 1150 °C for 15 min, after which they were quenched in a heated steel mold. To remove mechanical/thermal stress, the glasses were annealed for 3 h at 420 °C (below the glass transition temperature). The glasses were subsequently cooled to room temperature (RT), and then cut and polished to ∼1 mm thick slabs for spectroscopic measurements. Glass samples were also quenched as glass cylinders and cut to a length (*L*) of about 2.54 cm for dilatometric measurements. Some pieces were crushed by mortar and pestle for powder XRD measurements while some grains were used for DSC.

**Table 1:** Glass Codes and Nominal Compositions of the 50P_2_O_5_-(49 – *x*)­BaO-1Nd_2_O_3_-*x*SnO (*x* = 0, 1.0, 3.0, 5.0, 7.0, 9.0 mol %) Glasses Synthesized

glass	P_2_O_5_ (mol %)	BaO (mol %)	Nd_2_O_3_ (mol %)	SnO (mol %)
Nd	50.0	49.0	1.0	—
1SnNd	50.0	48.0	1.0	1.0
3SnNd	50.0	46.0	1.0	3.0
5SnNd	50.0	44.0	1.0	5.0
7SnNd	50.0	42.0	1.0	7.0
9SnNd	50.0	40.0	1.0	9.0

### 2.2 Measurements

Powder XRD characterization was performed at RT to confirm the amorphous nature of the glasses (crushed by mortar and pestle) with a PANalytical Empyrean X-ray diffractometer using Mo-K_α_ radiation (λ = 0.71 Å). The acceleration voltage used was 60 kV and the current 40 mA.

^119^Sn Mössbauer spectroscopy measurements were carried out using a M6 Resonant Gamma-ray Spectrometer (SEE Co.) with a Kr/CO_2_ proportional counter and a ^119m^Sn/CaSnO_3_ radioisotope source (Ritverc). All measurements were obtained with the sample and source at RT. The velocity axis was calibrated using a ^57^Co/Rh radioisotope source and a 25 μm thick α-Fe reference foil (Ritverc), and all isomer shifts are given relative to a natural abundance SnO_2_ powder at 0 mm/s.

Glass densities were determined by the Archimedes principle using distilled water as immersion liquid at RT in a Mettler-Toledo XSR Analytical Balance. The measurements were done in triplicate and the averages are reported (uncertainties in third decimal place). Various physical parameters that were useful for characterizing the glasses were then calculated by use of corresponding formulas.^11,14^

Raman spectroscopy measurements were carried out on the polished glass slabs at RT with a Thermo Scientific DXR Raman microscope (532 nm laser with power at 10 mW). The instrument was calibrated using a proprietary autoalignment tool and a polystyrene film prior to data collection. The data was collected using the 10× MPlan objective with an acquisition time for each spectrum of 100 s. Baseline subtraction was then done using OriginPro and the spectra normalized for comparison.

DSC was carried out in a SDT650 calorimeter (TA Instruments) in alumina pans under nitrogen gas atmosphere (flow rate at 100 mL/min) at a heating rate of 10 °C/min. The thermal parameters of interest [glass transition temperature (*T*_g_), onset of crystallization (*T*_*x*_), peak crystallization temperature (*T*_c_)] were estimated using the instrument’s software (midpoint-inflection point for *T*_g_).

Dilatometry measurements were carried out for the ∼2.54 cm-long glass cylinders at a heating rate of 3 °C/min in an Orton dilatometer (model 1410B). The thermal parameters of interest such as the coefficient of thermal expansion (CTE), *T*_g_ and the softening temperature (*T*_s_) were then estimated through the instrument’s software.

UV–vis–NIR spectrophotometry measurements were made on the ∼1 mm thick glass slabs taken at RT with an Agilent Cary 5000 double-beam spectrophotometer; air was always the reference.

The collection of PL emission and excitation spectra was done under static conditions (1 nm step size) at RT using a Horiba Fluorolog-QM spectrofluorometer with a Xe lamp. Emission decay curves were recorded using as excitation source a Xe flash lamp (∼2 μs pulse duration). The InGaAs detector was used for NIR measurements.

## 3 Results and Discussion

### 3.1 XRD

Shown in Figure 1 are the XRD patterns obtained for the Nd and 1-9SnNd glasses using Mo-K_α_ radiation (λ = 0.71 Å). The diffractograms show broad features toward small 2θ values characterizing long-range structural disorder.^11,14^ Some intensity fluctuations are seen with the broad humps, yet the diffractograms do not show discrete crystallization peaks. The XRD results thus support that amorphous solids were realized for the set of glasses within the range of SnO concentrations considered.

**Figure 1:**
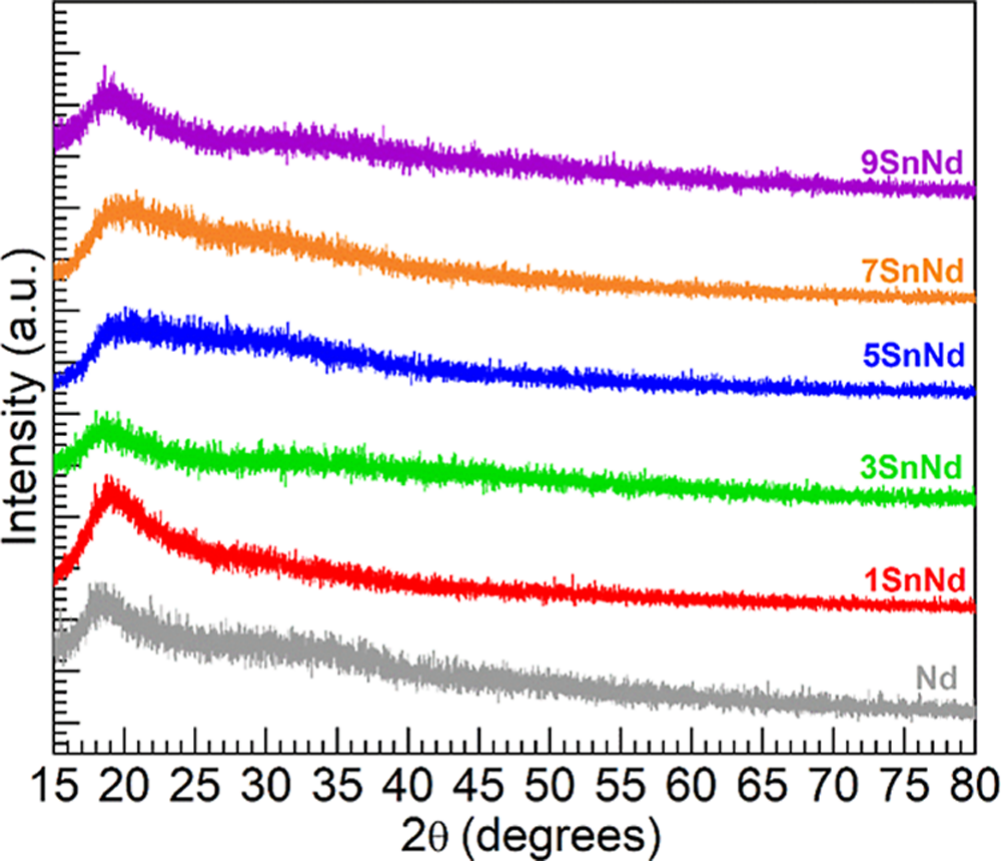
XRD patterns obtained for the various glasses with Mo-K_α_ radiation (λ = 0.71 Å).

### 3.2 ^119^Sn Mössbauer Spectroscopy

Prior to the evaluation of the structural, thermal and optical properties of the glasses, we proceed at this point with tin speciation analysis by ^119^Sn Mössbauer spectroscopy. The spectra obtained for the 1-9SnNd glasses are shown in Figure 2a–e. It is noticed that all samples show singlets near 0 mm/s indicative of Sn^4+^, while the presence of Sn^2+^ is evidenced by the quadrupolar doublets spanning 1–6 mm/s.^23,34,38,39^ The data thus shows that some SnO oxidation occurred for all glasses during the melting. This is in fact expected given that the synthesis was carried out under ambient atmospheric conditions, as similarly reported in various works.^26,28,34,37^ To estimate the relative amounts of Sn^2+^ and Sn^4+^, peak deconvolution was performed on the spectra as previously reported.^34^ While the Sn^2+^ doublet could be fit with two Lorentzian lines of equal width, the Sn^4+^ singlet was best fit with a Gaussian line shape; the fits were not improved any further using pseudo-Voigt lineshapes, so the minimal fitting model of one Gaussian and two Lorentzians was used for all spectra. The results are shown as the dashed traces in Figure 2a–e, where the cumulative fits are presented as solid traces. The relative percentage of Sn^2+^ in each glass was estimated from the prefactors of the normalized lineshapes obtained from these fits, and the values are shown in the respective panels of Figure 2 for quick access. The complete spectral parameters and estimated percent abundances of Sn^2+^ and Sn^4+^ deduced for each glass are presented in Table 2. It is noticed that even though Sn^4+^ occurs in all glasses, most of the tin exists as divalent tin. The relative Sn^2+^ percent estimated for the 9SnNd glass of 85.5% is close to the 88% reported for a phosphate glass made similarly with 2 mol % Yb_2_O_3_ and 10 mol % SnO (each added in relation to P_2_O_5_) as also estimated by ^119^Sn Mössbauer spectroscopy.^34^ Reasonable agreement is also observed with other reported barium phosphate glasses made similarly with 10 mol % additive SnO and containing Eu_2_O_3_ ([Sn^2+^] = 73.2%^26^) or Pr_2_O_3_ ([Sn^2+^] = 81.6%^28^) as appraised by X-ray photoelectron spectroscopy. Thus, in the presence of merely trivalent rare earth oxides, the oxidation of SnO appears to be essentially driven by the oxidizing air atmosphere. This contrasts with the situation with Tb_4_O_7_, which was observed to exert an additional oxidizing effect on SnO.^37^ Herein, the Nd_2_O_3_ employed, which was constant in all glasses at 1 mol %, is not expected to promote such an oxidizing effect.

**Figure 2:**
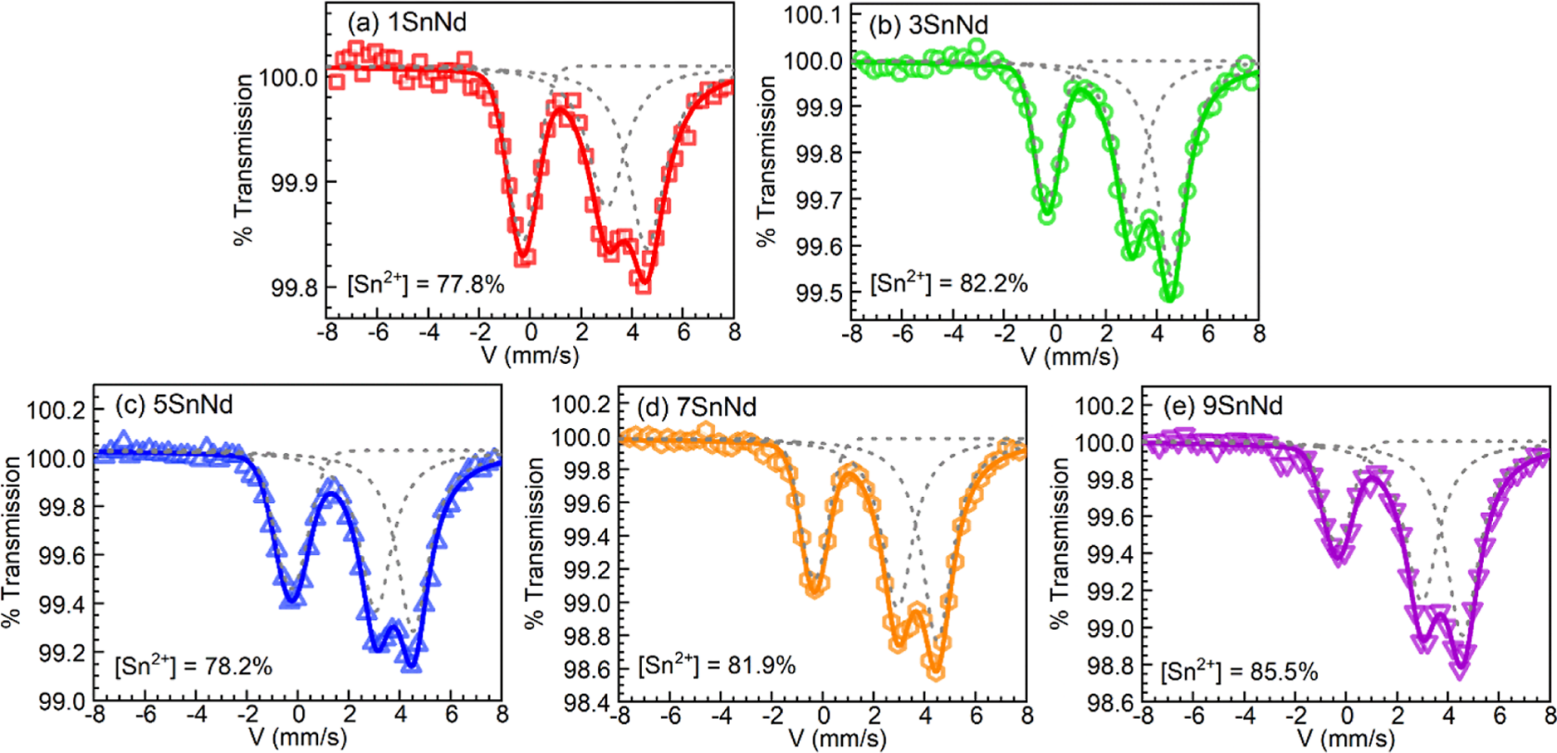
^119^Sn Mössbauer spectra (open symbols) for the tin-containing glasses: (a) 1SnNd; (b) 3SnNd; (c) 5SnNd; (d) 7SnNd; and (e) 9SnNd. The corresponding deconvolutions employed for tin speciation are the dashed traces overlaid; the solid traces are the cumulative fits. The spectral parameters and estimated percent abundances of Sn^2+^ and Sn^4+^ deduced for each glass are summarized in Table 2.

**Table 2:** Isomer Shift, Quadrupole Splitting, Line Width, and % Abundance of the Tin Species in the 1-9SnNd Glasses as Estimated from ^119^Sn Mössbauer Spectra

		^119^Sn Mössbauer parameter			
glass	component	isomer shift velocity (mm/s)	quadrupole splitting velocity (mm/s)	linewidth (mm/s)	% abundance
1SnNd	Sn^2+^	3.79	1.58	1.73	77.8
	Sn^4+^	–0.29	0	0.57	22.2
3SnNd	Sn^2+^	3.78	1.58	1.51	82.2
	Sn^4+^	–0.24	0	0.49	17.8
5SnNd	Sn^2+^	3.79	1.46	1.51	78.2
	Sn^4+^	–0.24	0	0.65	21.8
7SnNd	Sn^2+^	3.71	1.55	1.55	81.9
	Sn^4+^	–0.33	0	0.53	18.1
9SnNd	Sn^2+^	3.77	1.58	1.55	85.5
	Sn^4+^	–0.34	0	0.57	14.5

Using the nominal amounts of SnO (Table 1) and the relative percentages of Sn^2+^ and Sn^4+^ obtained from the ^119^Sn Mössbauer spectroscopy results (Table 2), the absolute concentrations of Sn^2+^ and Sn^4+^ in mol % in the glasses were estimated. The results are shown in Table 3. It is observed that the concentration of Sn^2+^ increases continuously in the glass series up to a maximum value of 7.7_0_ mol % Sn^2+^ for the 9SnNd glass with nominally 9.0 mol % SnO. On the other hand, the concentration of Sn^4+^ increases but reaches a maximum of ≈1.3 mol % in the 7SnNd and 9SnNd glasses. The estimated concentrations of the different tin species is an important aspect to consider in the interpretation of the various physicochemical properties herein assessed (vide infra). Meanwhile, the estimated concentrations of Sn^2+^ in mol % become useful in the evaluation of physical parameters following the assessment of glass densities considered next.

**Table 3:** Concentrations of Sn^2+^ and Sn^4+^ Estimated for the 1-9SnNd Glasses Based on Mössbauer Spectroscopy Analysis (Table 2) and the Nominal Concentrations of SnO (Table 1)

glass	Sn^2+^ (mol %)	Sn^4+^ (mol %)
1SnNd	0.78	0.22
3SnNd	2.4_7_	0.53
5SnNd	3.9_1_	1.0_9_
7SnNd	5.7_3_	1.2_7_
9SnNd	7.7_0_	1.3_1_

### 3.3 Density and Basic Physical Properties

Table 4 summarizes the densities obtained for the Nd and 1-9SnNd glasses along with other physical quantities calculated. The density of the Nd glass is the lowest at 3.674 g/cm^3^, yet there is no trend afterward for the 1-9SnNd glasses, as the values fluctuate. This suggests that differences arise due to experimental variability. The molar masses of SnO (134.71 g/mol) and BaO (153.33 g/mol) are close, with the former having a somewhat lower value. All else the same, the substitution of BaO by SnO should lead to slightly lower density values. However, the analysis of the ^119^Sn Mössbauer spectra above indicated that SnO oxidized to some extent in the 1-9SnNd glasses. This could be interpreted as a partial conversion of SnO (134.71 g/mol) to SnO_2_ (150.71 g/mol) with molar mass similar to that of BaO (153.33 g/mol). In this context, the fluctuating density values obtained for the 1-9SnNd glasses may reflect several experimental factors such as oxygen uptake from air accompanying the SnO oxidation, i.e.

$$SnO+\frac{1}{2}O_{2}\to {SnO}_{2}$$ 1

and changes in molar volumes (*V*_m_) of the glasses. Hence, to assess the latter parameter, *V*_m_, we proceed first to calculate the average molar mass (*M*_av_) of each glass by considering that the amounts of Sn^2+^ and Sn^4+^ (mol %) estimated from the ^119^Sn Mössbauer analysis (Table 3) have associated equivalent amounts of SnO and SnO_2_. The *M*_av_ values were then calculated from the following equation^11,14^

2 $$M_{av}=\sum_{i}X_{i}M_{i}$$

where *X*_*i*_ and *M*_*i*_ are the mole fractions and molar masses of each component, respectively, with the mole fractions of SnO and SnO_2_ being determined from the ^119^Sn Mössbauer results. From the measured densities (ρ), the molar volumes were obtained from the known formula^11,14^

3 $$V_{m}=\frac{M_{av}}{\rho }$$

**Table 4:** Densities and Other Parameters Related to the Physical Properties of the Different Glasses

parameter	Nd	1SnNd	3SnNd	5SnNd	7SnNd	9SnNd
density, ρ (g/cm^3^)	3.674	3.718	3.696	3.694	3.709	3.700
average molar mass, *M*_av_ (g/mol)	149.47	149.32	148.99	148.71	148.37	148.00
molar volume, *V*_m_ (cm^3^/mol)	40.68	40.16	40.31	40.26	40.01	39.99
Nd^3+^ concentration, *N*_Nd_ (×10^20^ ions/cm^3^)	2.960	2.999	2.988	2.992	3.011	3.011
Nd^3+^–Nd^3+^ mean distance, *d*_Nd–Nd_ (Å)	15.00	14.94	14.96	14.95	14.92	14.92
Sn^2+^ concentration, *N*_Sn_ (×10^20^ ions/cm^3^)	—	1.167	3.683	5.848	8.630	11.586
Sn^2+^–Sn^2+^ mean distance, *d*_Sn–Sn_ (Å)	—	20.47	13.95	11.96	10.50	9.52
Sn^2+^–Nd^3+^ mean distance, *d*_Sn–Nd_ (Å)	—	13.58	11.44	10.42	9.51	8.82

The *M*_av_ and *V*_m_ values are shown in Table 4 along with the densities. It is seen that the average molar masses decrease continuously, although only slightly, within the Nd and 1-9SnNd glass series. Hence, the 9SnNd glass exhibited the lowest *M*_av_ at 148.00 g/mol. The marginally decreasing trend thus follows with the prevalence of SnO in the glasses over SnO_2_. However, fluctuations are again observed regarding the molar volumes where the 7SnNd and 9SnNd glasses exhibited the lowest values at 40.01 and 39.99 cm^3^/mol, respectively.

We continue by calculating the concentration of optically relevant ions (e.g., luminescent) Nd^3+^ (*N*_Nd_) and Sn^2+^ (*N*_Sn_) in the glasses from the following equation^11,14^

4 $$N_{i}=\frac{{X_{i}\times \rho \times N}_{A}}{M_{av}}$$

using the corresponding mole fractions (*X*_*i*_), the glass densities (ρ), the average molar masses (*M*_av_), and Avogadro’s constant (*N*_A_). Here again, the mole fractions of Sn^2+^ were determined employing the ^119^Sn Mössbauer results. Then the mean distances between Nd^3+^ ions (*d*_Nd–Nd_) and the mean distances between Sn^2+^ ions (*d*_Sn–Sn_) were calculated from the individual ionic concentrations (*N*_*i*_) as^11,14^

5 $$d_{i-i}={\left(\frac{1}{N_{i}}\right)}^{\frac{1}{3}}$$

Lastly, the mean distances between Sn^2+^ and Nd^3+^ ions (*d*_Sn–Nd_) were determined from the following equation^14^

6 $$d_{i-j}={\left(\frac{1}{N_{i}+N_{j}}\right)}^{\frac{1}{3}}$$

where *N*_*i*_ and *N*_*j*_ are the respective ion concentrations. All the obtained values for the different glasses as applicable are presented in Table 4. The Nd^3+^ concentrations, *N*_Nd_, vary only slightly within the 2.960–3.011 × 10^20^ ions/cm^3^ range in accord with changes in the molar volumes, as the Nd_2_O_3_ content was fixed at 1.0 mol %. Accordingly, the mean Nd^3+^–Nd^3+^ distances, *d*_Nd–Nd_, fluctuate only marginally within the 15.00–14.92 Å range throughout the entire glass set, as expected. In relation to divalent tin concentration, *N*_Sn_, it increases continuously in the 1-9SnNd glasses within the 1.167–11.586 × 10^20^ ions/cm^3^ range. Hence, we see that the interionic distances, *d*_Sn–Sn_, decrease steadily from 20.47 to 9.52 Å. Finally, the Sn^2+^–Nd^3+^ mean distances, *d*_Sn–Nd_, are similarly observed to become shorter with the increase in tin content in the 1-9SnNd glasses, decreasing from 13.58 to 8.82 Å. These various parameters will become relevant for the discussion and interpretation of the luminescence results later considered (vide infra).

### 3.4 Raman Spectroscopy Assessment

Shown in Figure 3 are the Raman spectra obtained for the Nd and 1-9SnNd glasses following baseline subtraction and normalization with respect to the strongest band for comparison. The different features observed are assigned in accordance with reports of similar glass compositions.^11,14,35,37,40,41^ Beginning at the low energy region, the tin-free Nd glass taken as reference presents a band around 686 cm^–1^ recognized as the in-chain symmetric stretching vibrations in P–O–P bridges, ν_s_(POP), in *Q* ^2^ tetrahedral units (PO_4_ tetrahedra with 2 bridging oxygens, BOs). The small feature observed around 1005 cm^–1^ is credited to the symmetric stretch, ν_s_(PO_3_^2–^), in nonbridging oxygens (NBOs) concerning *Q* ^1^ units (PO_4_ tetrahedra with 1 BO). Then the Nd-doped glass shows the most intense band at about 1160 cm^–1^ due to the out-of-chain symmetric stretch in PO_2_^–^ groups, ν_s_(PO_2_^–^), occurring with the NBOs of the *Q* ^2^ units. Last of all, the corresponding asymmetric stretching vibrations, ν_as_(PO_2_^–^), are seen in the Nd glass around 1244 cm^–1^.

**Figure 3:**
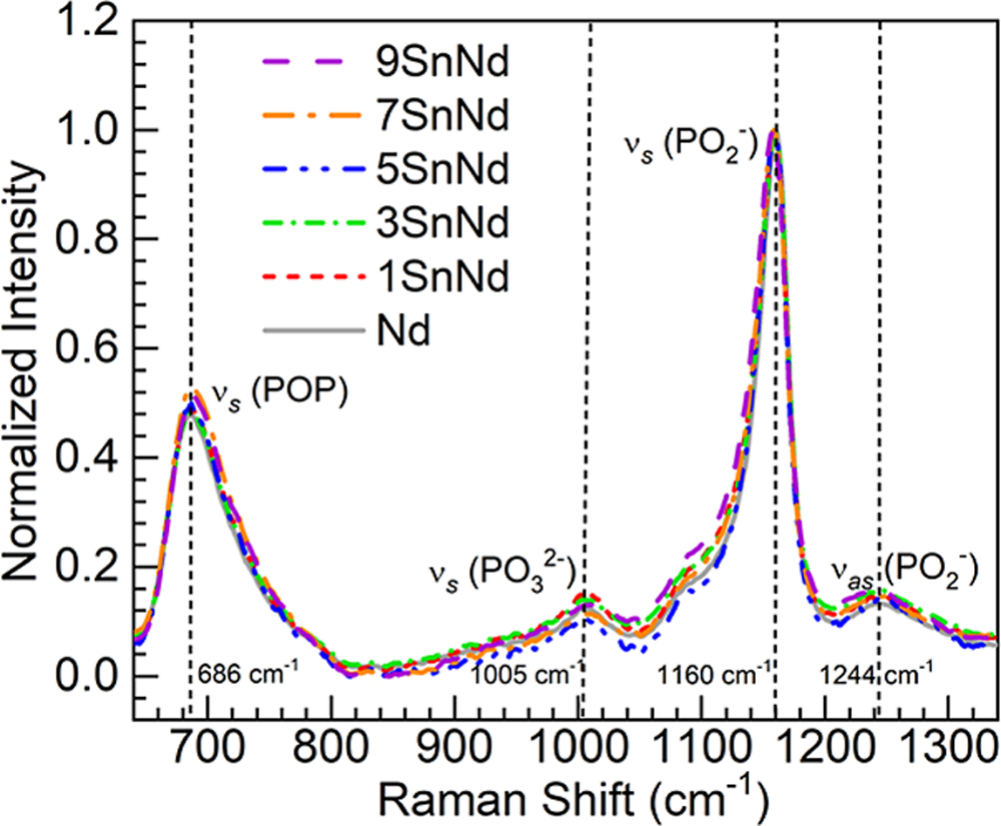
Raman spectra obtained for the Nd and 1-9SnNd glasses (normalized); main spectroscopic features in the Nd glass as reference are indicated (vertical dashed lines—frequencies displayed).

The Raman spectra for the 1-9SnNd glasses in Figure 3 appear very similar to the spectrum of the Nd glass. This suggests a lack of significant structural alteration upon substituting BaO by SnO in the codoped glasses. To assist with the evaluation, shown in Table 5 are the peak positions and full width at half-maximum (fwhm) values of the ν_s_(POP) band (BO-related) as well as the ν_s_(PO_2_^–^) band (NBO-related).^37,40^ Both bands exhibit a slight broadening with tin content, whereas variation in band frequencies are only minor. Overall, the data seems to support marginal depolymerization with the increase in tin content. This contrasts with the case where the rare earth codopant along with SnO was Tb_4_O_7_, which was observed to exert an oxidizing effect on SnO.^37^ There, the presence of Sn^4+^ was linked to the more clearly exhibited depolymerization effect.^37^ Hence, it appears in the present case that the minor changes in the Raman spectra of the 1-9SnNd glasses are induced by the relatively small amounts of Sn^4+^ ascertained (Table 3), which can act as a network modifier to produce a slight structural disruption.^37,42^

**Table 5:** Spectral Positions and Full Width at Half Maximum (FWHM) Values for the Bridging Oxygen (BO)-ν_s_(POP) and Non-Bridging Oxygen (NBO)-ν_s_(PO_2_^–^) Raman Bands Obtained for the Nd and 1-9SnNd Glasses

	BO-ν_s_(POP) band		NBO-ν_s_(PO_2_^–^) band	
glass	peak (cm^–1^)	fwhm (cm^–1^)	peak (cm^–1^)	fwhm (cm^–1^)
Nd	686	60	1160	31
1SnNd	687	62	1159	33
3SnNd	687	61	1159	33
5SnNd	687	62	1159	32
7SnNd	688	63	1158	33
9SnNd	688	63	1158	36

### 3.5 Thermal Properties

Figure 4 shows the DSC thermograms obtained for the different glasses under study. From these, the parameters of glass transition temperature, *T*_g_, onset of crystallization, *T*_*x*_, and peak crystallization temperature, *T*_c_, were estimated for each glass and are presented in Table 6. Added to Table 6 is the parameter indicating thermal stability, Δ*T* = *T*_*x*_ – *T*_g_.^14,37^ The tin-free Nd glass taken as reference exhibited a *T*_g_ at about 499 °C close to that reported for the undoped barium phosphate host of 497 °C.^11^ A crystallization peak was detected for the Nd glass with *T*_c_ around 710 °C and an onset, *T*_*x*_, at 670 °C. The resulting thermal stability factor Δ*T* is then 171 °C, which represents an improvement over the un-doped host with Δ*T* reported at 148 °C.^11^ It is noticed in Table 6 that the temperatures for all parameters are higher for the 1SnNd glass relative to the Nd reference. Especially noticeable is the increased *T*_g_ estimated for the 1SnNd glass at 508 °C. An upward trend in the *T*_g_ has been indicated to follow the increase in Sn^4+^ concentration (ascertained from X-ray absorption near-edge spectroscopy) in the DSC evaluation reported for barium phosphate glasses melted with SnO and Tb_4_O_7_.^37^ The shift in the glass transition to higher temperatures is likely due to the high field strength of Sn^4+^ ions acting as network modifiers.^43^ It then seems that the higher *T*_g_ of the 1SnNd glass is related to the presence of Sn^4+^ determined by ^119^Sn Mössbauer spectroscopy (Figure 2, Tables 2 and 3). On the other hand, the values of *T*_g_ and *T*_*x*_ exhibit decreasing trends for the 3-9SnNd glasses, and *T*_c_ and Δ*T* are seen to fluctuate. The 9SnNd glass shows the lowest *T*_g_ at 463 °C but the highest thermal stability Δ*T* = 191 °C. The decreasing trend in *T*_g_ is contrary to the leading effect of Sn^4+^ ions^37^ and thus points to a more significant impact from Sn^2+^ contents evidenced to be significant for the 3-9SnNd glasses by the ^119^Sn Mössbauer analysis (Table 3). This concurs with other works in ternary phosphate glasses, wherein a decrease in *T*_g_ values has been observed with increasing SnO content.^21,22^

**Figure 4:**
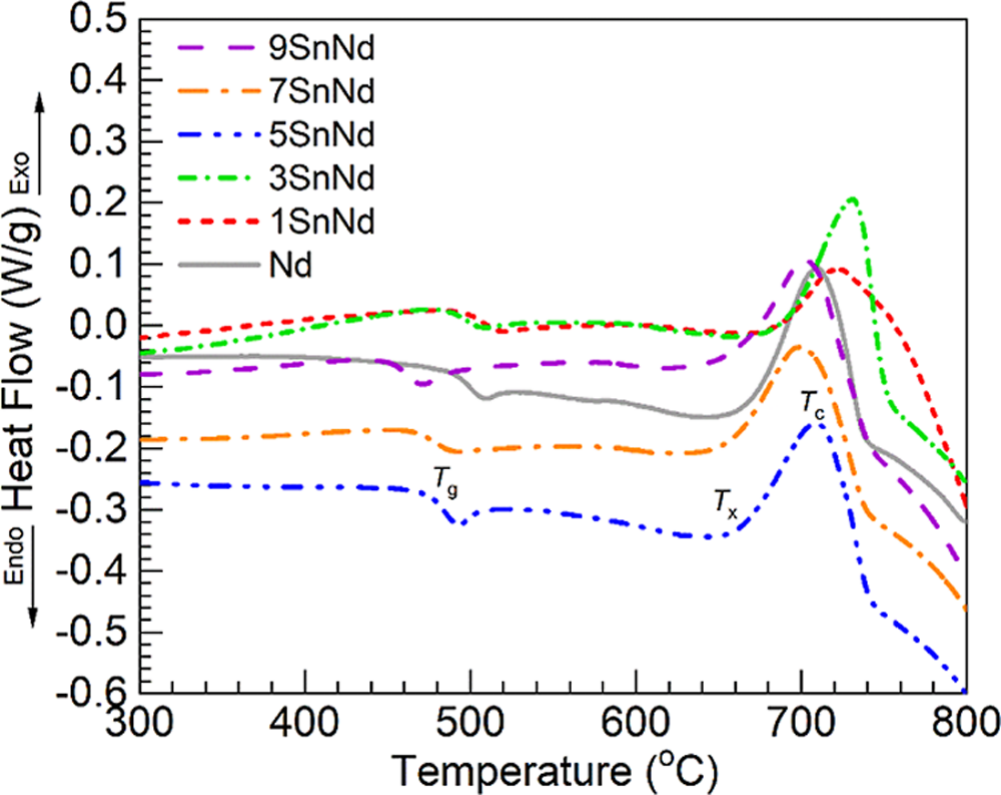
DSC thermograms obtained for the Nd and 1-9SnNd glasses displaying the regions of glass transition temperature (*T*_g_), onset of crystallization (*T*_*x*_), and crystallization temperature (*T*_c_); regions indicated for the 5SnNd glass (estimated values for all glasses presented in Table 6).

**Table 6:** Glass Transition Temperature (*T*_g_), Onset of Crystallization (*T*_*x*_), Main Peak Crystallization (*T*_c_) Temperature, and Thermal Stability Parameter Δ*T* = *T*_*x*_ – *T*_g_, Estimated for the Nd and 1-9SnNd Glasses from the DSC Profiles

glass	*T*_g_ (°C)	*T*_*x*_ (°C)	*T*_c_ (°C)	Δ*T* = *T*_*x*_ – *T*_g_ (°C)
Nd	499	670	710	171
1SnNd	508	684	723	176
3SnNd	499	687	732	188
5SnNd	485	661	711	176
7SnNd	478	653	701	175
9SnNd	463	654	704	191

We continue with evaluating the thermomechanical properties of the various glasses by means of the dilatometry data presented in Figure 5. The linear expansion regimes of the different curves appear similar for the different glasses. The main differences are noticed at high temperatures where the glasses go through the glass transition region and reach the peak dilatometric temperature. The thermal expansion profiles shown in Figure 5 were then used to estimate *T*_g_ as well as the parameters of dilatometric or softening temperature, *T*_s_, and the coefficient of thermal expansion, CTE, which was evaluated in the 50–400 °C range.^11^ The results for the different parameters obtained are presented in Table 7. It is noticed that the *T*_g_ values are somewhat lower than those obtained from DSC (Table 6), which is not uncommon.^37,44^ However, the type of behavior seen in the DSC results (Table 6) is similarly observed in the dilatometry results (Table 7). The *T*_g_ of the 1SnNd glass of 490 °C is higher than that of the Nd reference estimated at 486 °C (Table 7), which points to the significant impact of Sn^4+^ ions, as similarly observed for glasses melted with SnO and Tb_4_O_7_.^37^ Krohn et al.^39^ performed a dilatometric analysis on a different tin-doped glass system, namely soda-lime silica glass, and consistently reported a noticeable influence by SnO_2_ for achieving a higher *T*_g_. Then, as seen in Table 7, the 3-9SnNd glasses exhibit a downward trend for the *T*_g_ values relative to the 1SnNd glass. Consistent with this is also the behavior observed for the *T*_s_ results in Table 7, which follow the same evolution as the *T*_g_, suggesting an overruling effect from Sn^2+^. It turns out that Krohn et al.^39^ also observed for tin-doped silica glasses doped with fixed tin oxide at 2 mol % that the *T*_g_ and *T*_s_ values decreased with increasing Sn^2+^ fraction. The data from the two different techniques herein thus harmonize and point to the noticeable effect of Sn^4+^ in the 1SnNd glass but a dominant influence from Sn^2+^ in the 3-9SnNd glasses that lowers the glass transition and softening temperatures.

**Figure 5:**
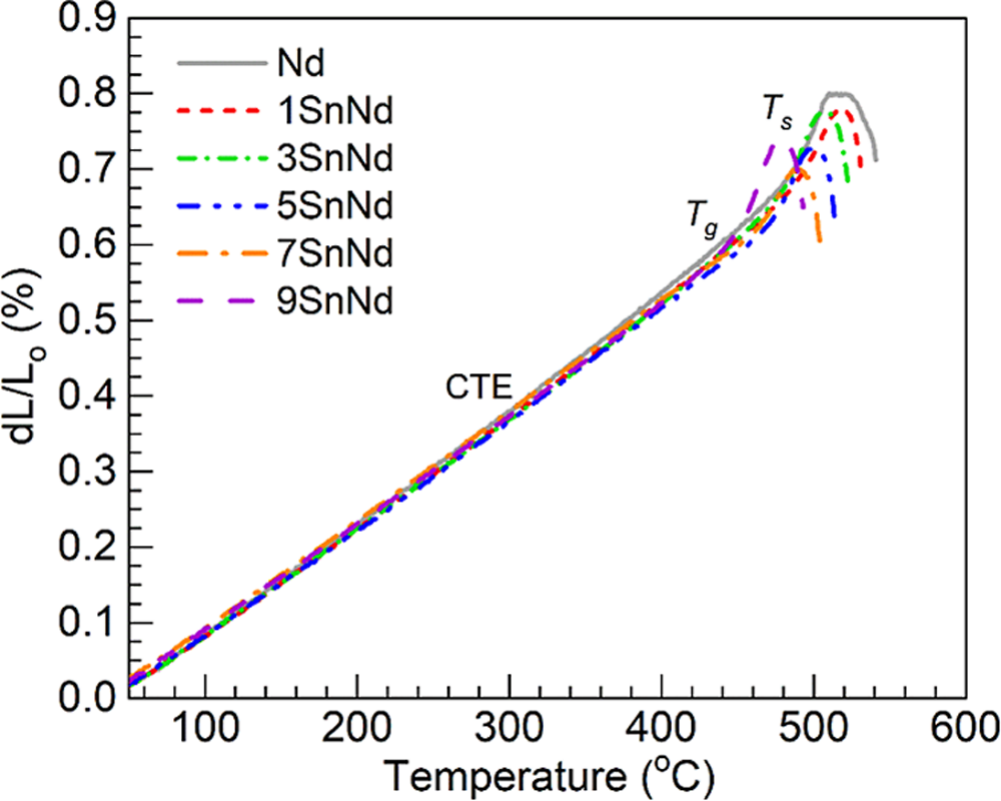
Dilatometric profiles obtained for the Nd and 1-9SnNd glasses; the estimated values of glass transition temperature (*T*_g_), softening temperature (*T*_s_) and coefficient of thermal expansion (CTE) are presented in Table 7.

**Table 7:** Values of Glass Transition Temperature (*T*_g_), Softening Temperature (*T*_s_) and Coefficient of Thermal Expansion (CTE, Estimated in the 50–400 °C Range) Obtained for the Different Glasses From Dilatometry

glass	*T*_g_ (°C)	*T*_s_ (°C)	CTE (×10^–6^ °C^–1^)
Nd	486	510	14.6
1SnNd	490	519	14.2
3SnNd	475	507	14.1
5SnNd	472	502	14.0
7SnNd	468	490	14.0
9SnNd	447	479	14.0

With respect to the CTE, it is interesting that the 1SnNd glass exhibits a value of 14.2 × 10^–6^ °C^–1^, a decrease compared to the Nd reference with CTE of 14.6 × 10^–6^ °C^–1^ (Table 7). This indicates that a somewhat tighter glass network was produced with 1 mol % SnO added in place of BaO. On the other hand, the CTE of the 3SnNd glass is estimated at 14.1 × 10^–6^ °C^–1^ and those of the 5-9SnNd glasses are equally estimated at 14.0 × 10^–6^ °C^–1^ (Table 7), all of which are more similar to that of the 1SnNd glass. Admittedly, disentangling the effects of the different oxidation states of tin on the thermal expansion of glasses is a challenging task. The present results at least do not contradict the dilatometric study of tin-doped silica glasses carried out by Krohn et al.,^39^ where it was noticed that the CTE values were inclined to decrease in a similar manner with both SnO and SnO_2_. However, a contrast is seen with the case of the phosphate glasses melted with SnO and Tb_4_O_7_, wherein a small amount of SnO produced an increase in the CTE relative to the Tb-doped tin-free glass.^37^ In that instance, Tb_4_O_7_ exerted a major impact on SnO speciation, leading to tin being present mostly as the Sn^4+^ ions linked to the decreased rigidity.^37^ Additionally, as more Sn^2+^ was incorporated in the Tb-doped glasses relative to Sn^4+^, the CTE values tended to decrease but were never as low as those obtained for the tin-free Tb-doped ref 37. However, the presence of Sn^4+^ in the glasses studied in ref 37 was also indicated to induce glass depolymerization. It is known that the varying extent of depolymerization in glasses can impact the CTE. For instance, a high degree of depolymerization is commonly linked to high CTE values in connection with looser glass networks.^45,46^ It is then likely that the relatively high CTE values reported in ref 37 reflected the effects of glass depolymerization in conjunction with the influence of tin in different oxidation states. In the present study, replacing BaO with SnO always produced a lower CTE, especially noticeable at low concentrations. In addition, the Raman spectroscopy analysis carried out did not support significant depolymerization, especially for the lowest SnO contents (Figure 3 and Table 5). Hence, in the present case the lower CTE in the 1SnNd glass could be linked to the lack of network depolymerization and the presence of the high field strength Sn^4+^ ions, which are also suggested to increase the *T*_g_ and *T*_s_ (vide supra). Thereafter, the increasing occurrence of Sn^2+^ (Table 3) did not appear to further decrease the CTE to a significant extent. The fact that the 5SnNd, 7SnNd and 9SnNd glasses had Sn^4+^ concentrations of 1.0_9_, 1.2_7_ and 1.3_1_ mol % (Table 3), respectively, is at least consistent with the lack of change in the CTE being related to similar quantities of Sn^4+^. Henceforth, we focus on evaluating the optical properties of the glasses, converging on the luminescent behavior highly impacted by divalent tin.

### 3.6 Optical Properties

The absorption spectra measured from the UV to the NIR for the different glasses are shown in Figure 6 along with sample photographs. The glasses presented similarly a light purple color in accord with the comparable Nd^3+^ concentrations contained. The spectra in Figure 6 in fact all display in like manner the different transitions characteristic of Nd^3+^ ions originating from the ^4^I_9/2_ ground state, most prominently the absorption peak around 583 nm linked to ^4^I_9/2_ → ^4^G_5/2_ + ^2^G_7/2_ transitions.^3,10,11^ That the spectra are analogous harmonizes with the fact that the concentration of Nd_2_O_3_ in all glasses was kept constant at 1 mol %, in clear contrast with cases in which the neodymium content is varied in the glasses.^11,14^ This upholds that the Nd and 1-9SnNd glasses had Nd^3+^ ions similarly incorporated. The incremental amounts of SnO added at the expense of BaO in the 1-9SnNd glasses have no distinct impact in the visible and NIR regions of the absorption spectra. This is because the Sn^2+^ centers absorb in the UV region, which is obscured by the absorption inherent to the glass host.^26,28,29,34,36^ The presence of the divalent tin centers however becomes evident through their PL properties, as considered next.

**Figure 6:**
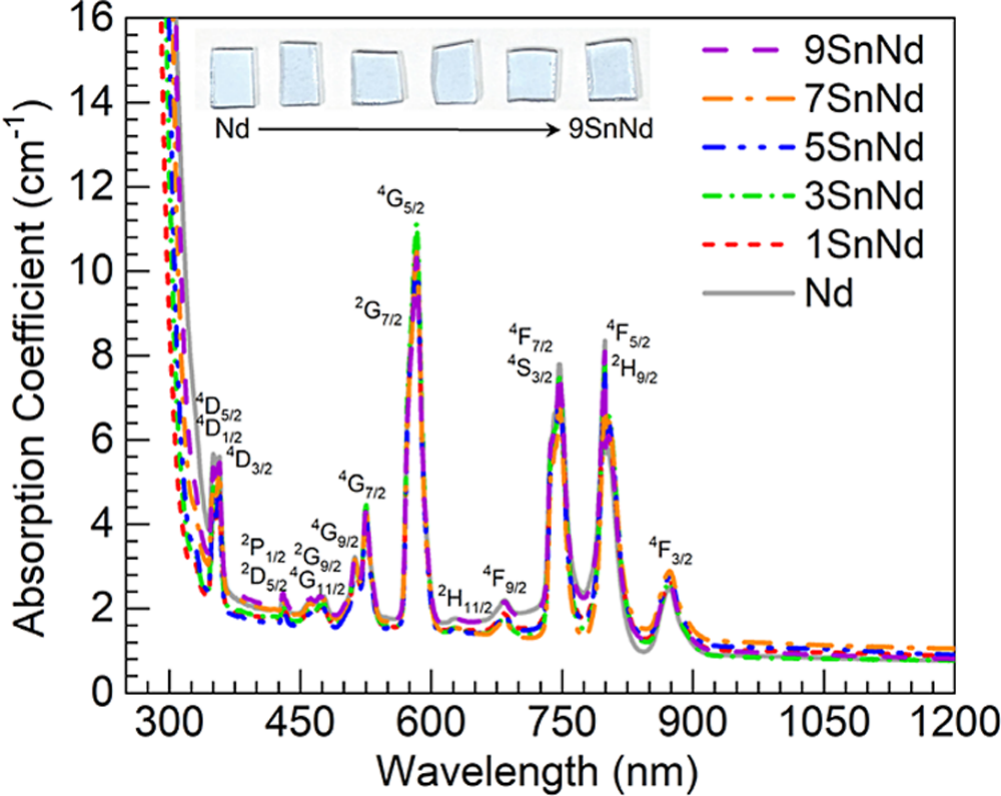
UV–vis–NIR absorption spectra obtained for the different glasses with some excited states associated with prominent absorption in Nd^3+^ ions from the ^4^I_9/2_ ground state indicated. The inset shows photographs of samples on a white background (from left to right: Nd, 1SnNd, 3SnNd, 5SnNd, 7SnNd and 9SnNd).

We continue with the PL investigation by considering excitation spectra obtained by monitoring the key emission from Nd^3+^ ions at 1056 nm due to the ^4^F_3/2_ → ^4^I_11/2_ lasing transition. The spectra recorded for all glasses are shown in Figure 7, where we focus on the UV region relevant to the excitation of divalent tin centers.^16,26–31,34–37^ It is observed that the tin-free Nd glass lacks any excitation features in the region being non-resonant for exciting Nd^3+^ ions between 220 and 320 nm. Conversely, a new excitation band emerges in the 1SnNd glass peaking around 270 nm. This type of band has been associated with the excitation of singlet-to-singlet transitions (*S*_0_ → *S*_1_) in Sn^2+^ centers commonly linked with 2-fold coordination in glasses.^16,28–31,34–37,47,48^ It is direct evidence of an energy transfer process leading to the sensitized emission from Nd^3+^ ions. This confirms the original work of Malashkevich et al.^15^ on the sensitization of Nd^3+^ PL by divalent tin and the more recent report from Bondzior and Lisiecki^16^ indicating a nonradiative interaction of the dipole–dipole type. It is also consistent with reports wherein divalent tin has been suggested to play a partial role in the enhancement of Nd^3+^ emission in phosphate glasses codoped with SnO/CuO^17,20^ and SnO/Ag_2_O.^18^ It is noticed in Figure 7 that the tin-related excitation band increases in intensity and broadens for the 3SnNd and 5SnNd glasses, ultimately shifting to about 280 and 290 nm, respectively. It then starts to be somewhat suppressed for the 7SnNd and 9SnNd glass while continuing to shift, displaying maxima around 295 and 300 nm, correspondingly. This behavior thus reflects the increase in Sn^2+^ concentration (Table 3), suggesting an optimum Nd^3+^ emission due to Sn^2+^ → Nd^3+^ energy transfer for the 5SnNd glass. The subsequent intensity decrease observed for the 7SnNd and 9SnNd glasses thereafter suggests the concentration self-quenching effect inherent to Sn^2+^ pointed out by Malashkevich et al.^15^

**Figure 7:**
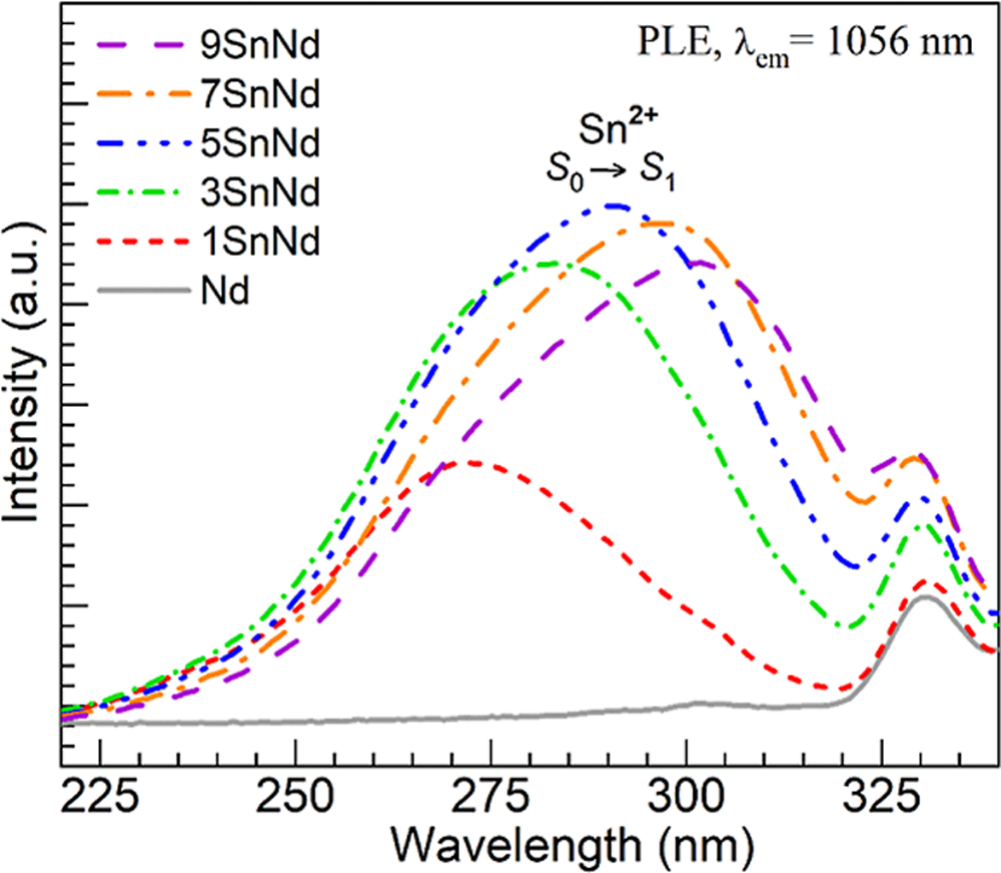
PL excitation (PLE) spectra obtained for the different glasses by monitoring NIR emission from Nd^3+^ ions at 1056 nm.

The evolution of the Nd^3+^ luminescence with tin content is validated by the NIR emission spectra shown in Figure 8. The emission spectra were obtained for the different glasses using the respective maximum peak excitation wavelengths detected in Figure 7 (indicated in parentheses next to each glass label in the legend in Figure 8). The tin-free Nd glass PL spectrum was recorded as reference under excitation at 270 nm. It displays rather weak emission given that the excitation wavelength is not resonant with any of the main Nd^3+^ ions absorption peaks. However, the 1-9SnNd glasses clearly exhibit the typical Nd^3+^ ^4^F_3/2_ → ^4^I_9/2_, ^4^I_11/2_, ^4^I_13/2_ NIR transitions.^10,11,14^ Here again, it is noticed that the PL intensity grows for the 3SnNd and 5SnNd glasses and then drops for the 7SnNd and 9SnNd glasses. Malashkevich et al.^15^ indicated that a PL quenching could take place for Nd^3+^ ions if the Sn^2+^ added to the glasses reduces the Fe^3+^ impurities to Fe^2+^, which is a known quencher of Nd^3+^.^49^ However, the Nd^3+^ PL evolution in Figure 8 is consistent with the excitation spectra in Figure 7 and points instead to the concentration quenching effect inherent to tin. In addition, emission decay curves were also obtained for the 1-9SnNd glasses by monitoring Nd^3+^ ^4^F_3/2_ → ^4^I_11/2_ emission at 1056 nm under excitation of the Sn^2+^ centers between 270 and 300 nm. The data are shown as semilog plots in Figure 9 with the excitation wavelengths used for each glass specified in the legend. The curves clearly exhibit exponential behavior and were therefore fit by a first-order exponential decay function^11,14^

7 $$I\left(t\right)=I_{0}\exp\left(-t/\tau \right)$$

where *I*(*t*) is the time-dependent luminescence intensity, *I*_0_ the initial intensity, and τ the excited state lifetime. The values obtained for the 1SnNd, 3SnNd, 5SnNd, 7SnNd and 9SnNd glasses were 231.1 (±0.2), 235.6 (±0.2), 234.1 (±0.2), 235.0 (±0.2) and 216.5 (±0.2) μs, respectively (values also shown in Figure 9). These lifetimes are comparable with those reported for similar glasses containing 1 mol % Nd_2_O_3_. For instance, a Nd^3+^ ^4^F_3/2_ lifetime of 212 (±1) μs was estimated under resonant Nd^3+^ excitation at 803 nm for the 50P_2_O_5_-49BaO-1Nd_2_O_3_ composition,^11^ whereas a value of 227 (±1) μs was deduced for 50P_2_O_5_-47BaO-2MnO-1Nd_2_O_3_ glass.^14^ Hence, significant production of Fe^2+^ impurities by Sn^2+^ does not seem to be the source of the Nd^3+^ quenching. The similarity of the lifetimes for 1-9SnNd relates to the comparable Nd^3+^-Nd^3+^ distances found for the glasses within 14.92–15.00 Å (Table 4). A concentration quenching effect, wherein high Nd^3+^ concentrations lead to interionic distances sufficiently short to allow excitation migration^11,14^ is then not likely. On the other hand, looking at Table 4 it is seen that the most favorable Sn^2+^ concentration was that for the 5SnNd glass of 5.355 × 10^20^ ions/cm^3^, with associated Sn^2+^–Sn^2+^ and Sn^2+^–Nd^3+^ mean distances of 12.32 and 10.62 Å, respectively. Although shorter Sn^2+^–Nd^3+^ distances were found for the 7SnNd and 9SnNd glasses (Table 4), which would be favorable for the Sn^2+^ → Nd^3+^ transfer, it seems that the accompanying shorter Sn^2+^–Sn^2+^ distances ultimately trigger a concentration quenching effect that becomes detrimental.

**Figure 8:**
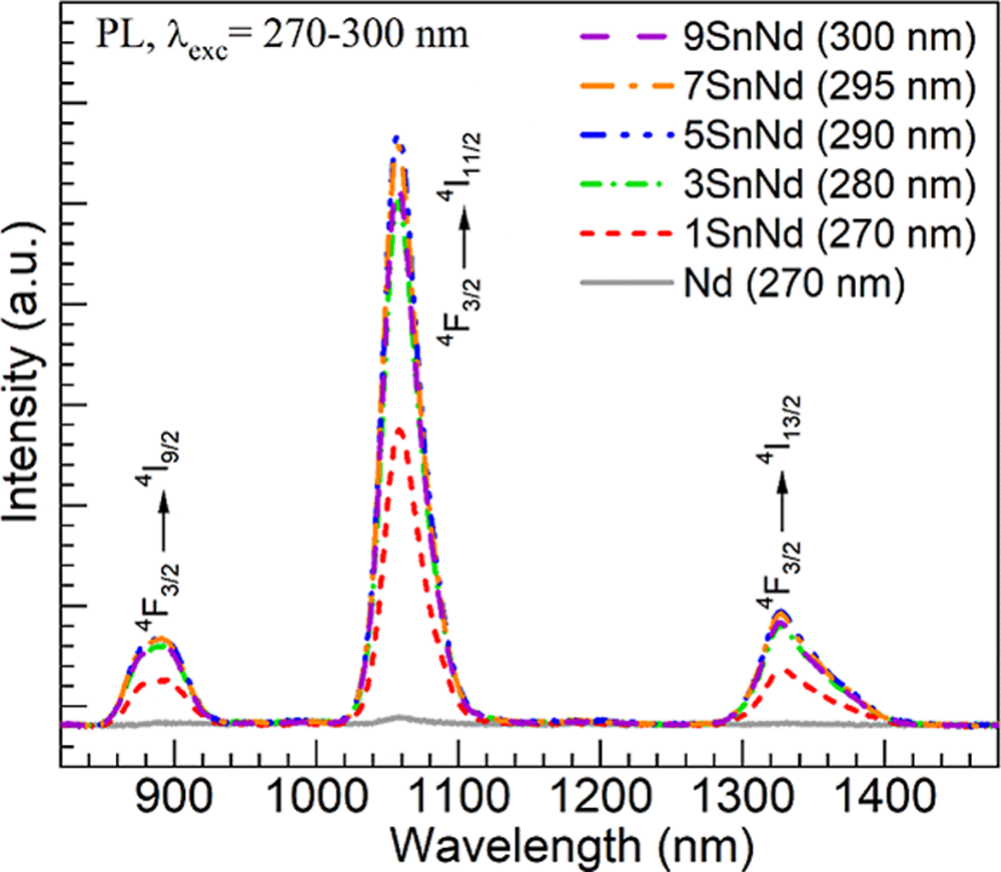
NIR emission spectra obtained for different glasses (excitation wavelengths indicated in parentheses next to each glass label in the legend).

**Figure 9:**
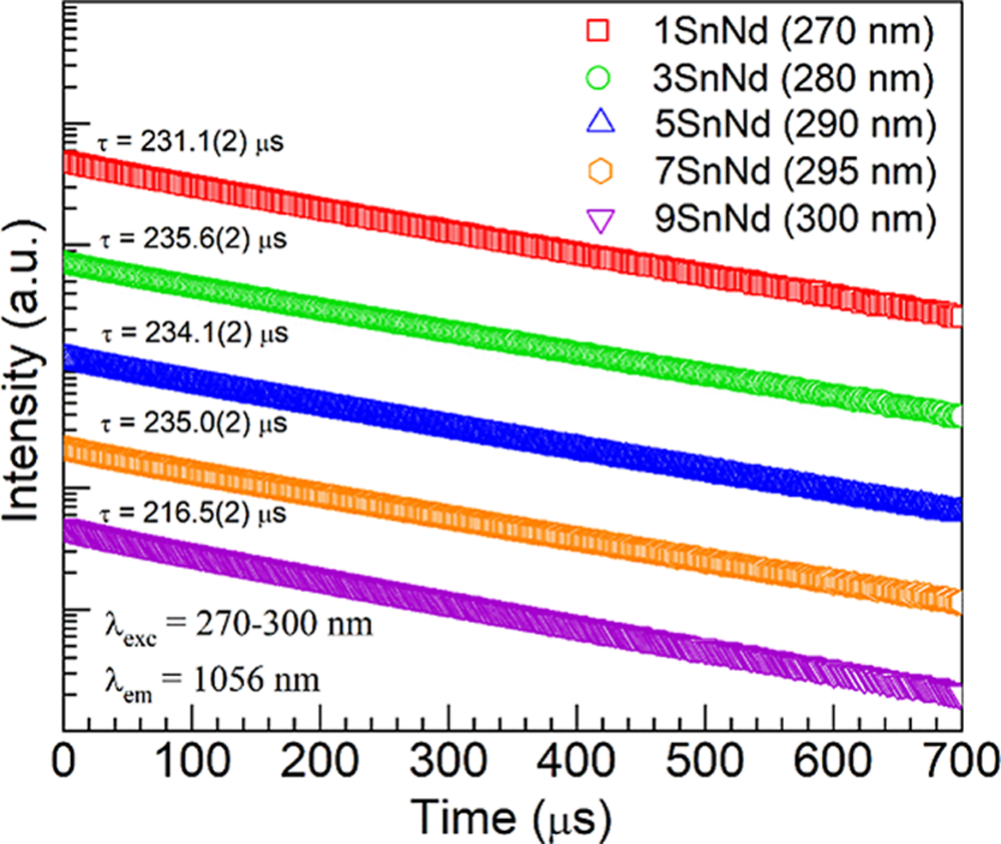
Semilog plots of the emission decay curves obtained for the 1-9SnNd glasses under excitation of divalent tin centers (excitation wavelengths used for each glass specified in the legend) while monitoring NIR emission at 1056 nm. The lifetimes estimated from single exponential fits are shown above each trace (the numbers in parentheses represent the uncertainties in the last digits).

In Figure 10, the visible emission stemming from divalent tin is assessed for the different glasses under the relevant excitations linked to *S*_0_ → *S*_1_ transitions in Sn^2+^ (the excitation wavelengths used for each glass are specified in the legend). The tin-free Nd glass was excited for reference purposes at 270 nm and again shows no significant emission, as expected. On the contrary, the 1SnNd glass shows a broad emission band with a maximum around 410 nm. This type of PL has been associated with the emission of triplet-to-singlet transitions (*T*_1_ → *S*_0_) in Sn^2+^ centers in glasses.^16,26,28–31,34–37^ The broad band intensifies and shifts slightly toward longer wavelengths for the 3SnNd and 5SnNd glasses and then diminishes for the 7SnNd and 9SnNd glasses. This behavior resembles that observed in the excitation spectra in Figure 7 and the NIR emission in Figure 8, thus supporting the tin-related concentration self-quenching effect.^15^ The spectra of the 1-9SnNd glasses in Figure 10 all show dips that correspond with Nd^3+^ absorption, as evidenced by the absorption spectrum of the Nd glass overlaid. This effect is proof of a resonant radiative energy transfer from the excited triplet states (*T*_1_) of divalent tin to the rare-earth ions.^17,20,31^ The nature of the Sn^2+^ → Nd^3+^ energy transfer was further investigated by Bondzior and Lisiecki^16^ through decay kinetic analyses in the context of the Inokuti-Hirayama model, indicating a nonradiative interaction of the dipole–dipole type. Herein, the processes involving Sn^2+^ and Nd^3+^ ions can be represented with the schematic diagram shown in Figure 11. Optical absorption (e.g., at 290 nm as with the 5SnNd glass) leads to the excitation of the singlet state (*S*_1_) in Sn^2+^ centers, after which intersystem crossing populates the emitting *T*_1_ states. The Sn^2+^ centers can then emit broadly (inhomogeneous broadening) as seen in Figure 10, which is illustrated in Figure 11 with 430 nm emission since it is also resonant with the ^2^P_1/2_ state in Nd^3+^ ions. Alternatively, the excited Sn^2+^ centers can transfer the energy to resonant energy levels in Nd^3+^ ions, such as the ^2^P_1/2_ state as illustrated in Figure 11. Besides the ^2^P_1/2_ state, other resonant states in Nd^3+^ likely involved are ^4^D_1/2_, ^4^D_3/2_, ^4^D_5/2_, ^2^D_5/2_, ^4^G_11/2_, ^2^G_9/2_, ^2^D_3/2_, ^2^P_3/2_, ^2^K_15/2_, ^4^G_9/2_, ^2^K_13/2_ and ^4^G_7/2_. Subsequently, these excited states then decay nonradiatively to the ^4^F_3/2_ metastable state, leading to the radiative relaxation in the NIR due to ^4^F_3/2_ → ^4^I_9/2_, ^4^I_11/2_, ^4^I_13/2_ transitions (Figure 8).

**Figure 10:**
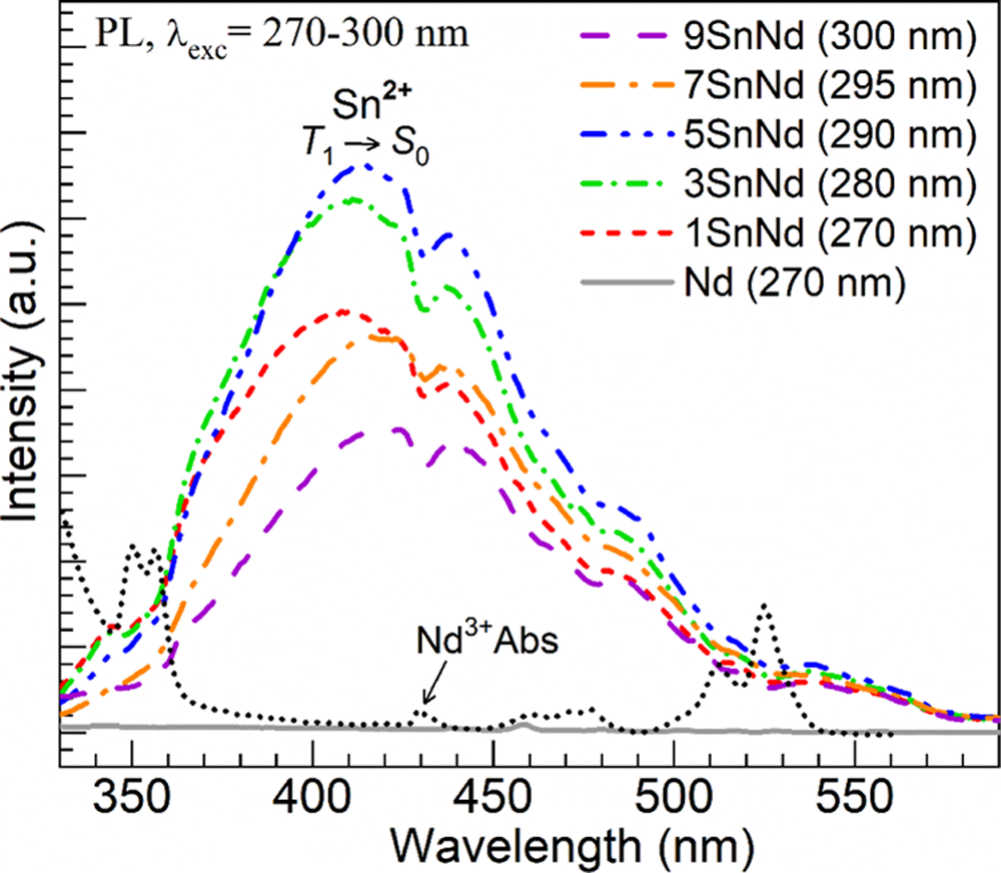
Visible emission spectra obtained for the different glasses (excitation wavelengths indicated in parentheses next to each glass label in the legend). The absorption spectrum of the Nd glass is overlaid to show the spectral overlap with absorption (Nd^3+^ Abs).

**Figure 11:**
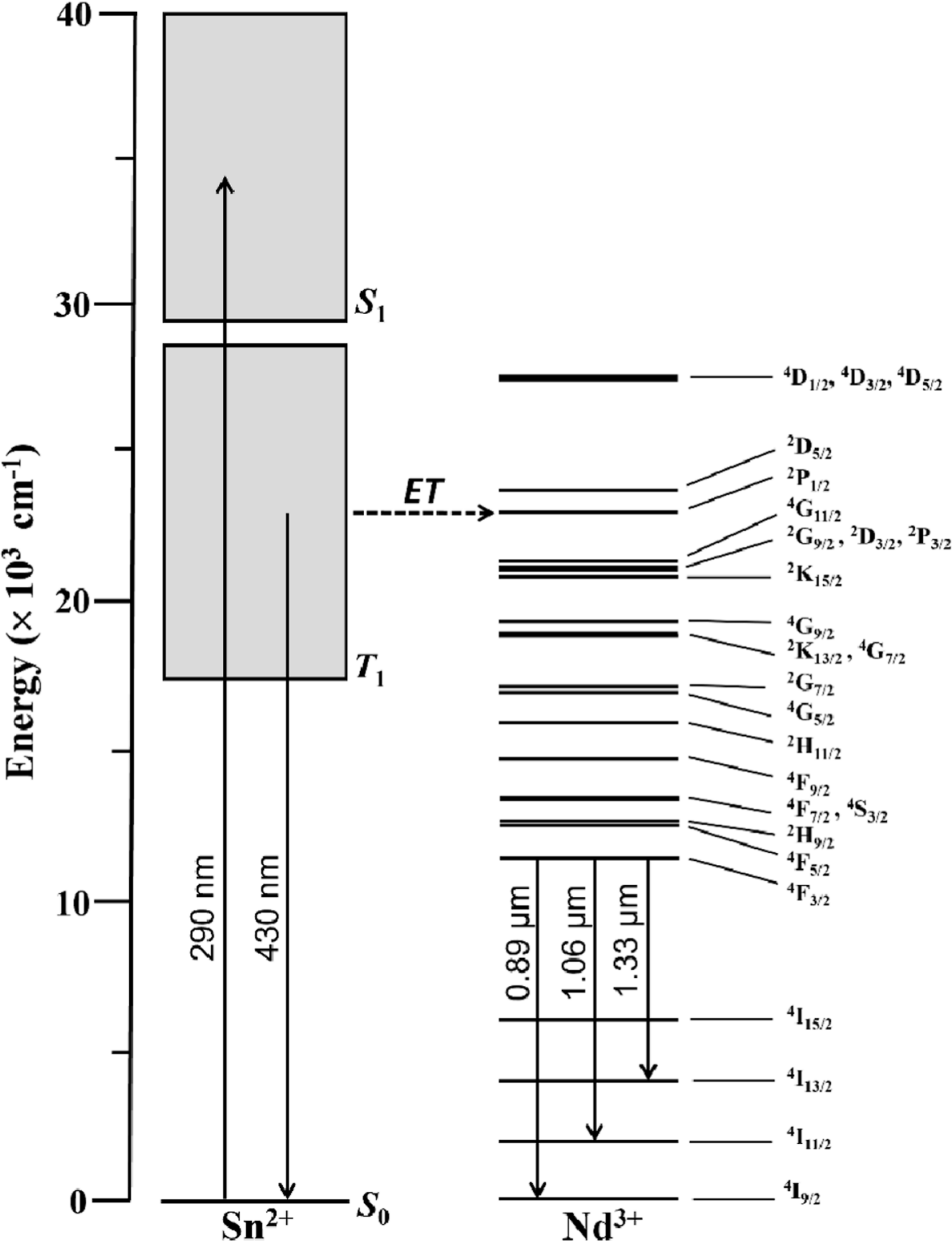
Simplified energy level diagram of Sn^2+^ and Nd^3+^ ions illustrating the excitation of the *S*_0_ → *S*_1_ transitions in Sn^2+^ at 290 nm (e.g., 5SnNd glass), the emission from the *T*_1_ state at 430 nm, and the energy transfer (ET) to the ^2^P_1/2_ resonant level in Nd^3+^ ions leading to the radiative transitions from the ^4^F_3/2_ emitting state (nonradiative relaxations omitted).

## 4 Summary and Conclusions

Recapitulating, glasses were prepared by the melt-quenching technique with 50P_2_O_5_-(49 – *x*)­BaO-1Nd_2_O_3_-*x*SnO (*x* = 0, 1.0, 3.0, 5.0, 7.0, and 9.0 mol %) compositions for a holistic study of various physicochemical properties, ultimately resulting in evaluation of the optimum conditions for the UV-excited Sn^2+^-sensitized NIR emission from Nd^3+^ ions. The investigation encompassed measurements by XRD, ^119^Sn Mössbauer spectroscopy, densitometry, Raman spectroscopy, DSC, dilatometry, optical absorption, and PL spectroscopy. The glasses were confirmed to be X-ray amorphous, whereas Raman spectroscopy showed no significant structural variation with SnO added at the expense of BaO. ^119^Sn Mössbauer spectroscopy was used for evaluating the speciation of tin in the glasses, showing that Sn^2+^ occurrence was favored even though some SnO oxidation occurred in the melts. The densities showed fluctuations which were rationalized in terms of SnO and SnO_2_ contents in correspondence with the Sn^2+^ and Sn^4+^ concentrations determined by ^119^Sn Mössbauer spectroscopy. Other physical parameters were then determined such as the Sn^2+^–Sn^2+^ and Sn^2+^–Nd^3+^ mean distances, which decreased with increasing nominal SnO concentrations. Thermal analyses by DSC and dilatometry suggested that Sn^2+^ was mostly influencing the glass transition and softening temperatures, showing a decreasing trend in the codoped glasses. However, the thermal expansion coefficients first decreased but then remained steady at high nominal SnO contents, likely connected with the lack of network depolymerization and the presence of relatively low amounts of Sn^4+^ ions with high field strength.

With respect to the optical properties, the absorption spectra appeared similar as expected for the fixed concentration of Nd_2_O_3_ of 1 mol % and the lack of influence from tin species in the visible and NIR regions. The PL evaluation then evidenced that exciting Sn^2+^ centers in the UV produced the sensitized NIR emission from Nd^3+^ ions. This was further observed to be maximized for SnO added at 5 mol % corresponding to about 3.9 mol % Sn^2+^ based on ^119^Sn Mössbauer spectroscopy. Higher tin concentrations were found to exhibit decreased intensities for the Nd^3+^ ^4^F_3/2_ → ^4^I_9/2_, ^4^I_11/2_, ^4^I_13/2_ NIR transitions, concurring with the evolution of Sn^2+^ PL. The Nd^3+^ lifetimes were evaluated and found to be comparable among the different samples. Accordingly, the presence of a significant quenching channel depopulating the ^4^F_3/2_ state in Nd^3+^ ions is not likely to produce the decreased NIR emission at high tin content. Instead, the concentration self-quenching effect inherent to Sn^2+^ ions is suggested to be the limiting factor in optimizing the nonresonant UV-excited NIR emission from Nd^3+^ ion via divalent tin centers.

## Data Availability

The data underlying this study are available in the published article and from the corresponding author upon reasonable request.
